# Blessings in disguise: a review of phytochemical composition and antimicrobial activity of plants belonging to the genus *Eryngium*

**DOI:** 10.1186/s40199-015-0136-3

**Published:** 2015-12-14

**Authors:** Sinem Aslan Erdem, Seyed Fazel Nabavi, Ilkay Erdogan Orhan, Maria Daglia, Morteza Izadi, Seyed Mohammad Nabavi

**Affiliations:** Department of Pharmacognosy, Faculty of Pharmacy, Ankara University, 06100 Ankara, Turkey; Applied Biotechnology Research Center, Baqiyatallah University of Medical Sciences, P.O. Box 19395-5487, Tehran, Iran; Department of Pharmacognosy, Faculty of Pharmacy, Gazi University, 06330 Ankara, Turkey; Department of Drug Sciences, Medicinal Chemistry and Pharmaceutical Technology Section, University of Pavia, Pavia, Italy; Health Research Center, Baqiyatallah University of Medical Sciences, Tehran, Iran

**Keywords:** Antibacterial, Eryngial, *Eryngium*, Flavonoids, Saponins, Infection

## Abstract

Medicinal and edible plants play a crucial role in the prevention and/or mitigation of different human diseases from ancient times to today. In folk medicine, there are different plants used for infectious disease treatment. During the past two decades, much attention has been paid to plants as novel alternative therapeutic agents for the treatment of infectious diseases due to their bioactive natural compounds such as phenol, flavonoids, tannins, etc. The genus *Eryngium* (Apiaceae) contains more than 250 flowering plant species, which are commonly used as edible and medicinal plants in different countries. In fact, some genus *Eryngium* species are used as spices and are cultivated throughout the world and others species are used for the treatment of hypertension, gastrointestinal problems, asthma, burns, fevers, diarrhea, malaria, etc. Phytochemical analysis has shown that genus *Eryngium* species are a rich source of flavonoids, tannins, saponins, and triterpenoids. Moreover, eryngial, one the most important and major compounds of genus *Eryngium* plant essential oil, possesses a significant antibacterial effect. Thus, the objective of this review is to critically review the scientific literature on the phytochemical composition and antibacterial effects of the genus *Eryngium* plants*.* In addition, we provide some information about traditional uses, cultivation, as well as phytochemistry.

## Background

Infectious diseases are known as one of the most important leading causes of long and short–term morbidity and mortality worldwide [1, 2]. According to the World Health Organization, in 2011, infectious diseases were responsible for approximately 18 million deaths worldwide. In addition to the high prevalence of infectious diseases, there are some microorganisms resistant to antibiotic therapy, which lead to the increase of death rate due their ability to acquire and transmit drug resistance [2]. Nowadays, antibiotic resistance is known as one the most important and challenging health problems in the global health programs. Therefore, during the past two decades, much attention has been paid to the discovery and development of natural multi-target antimicrobial agents with high efficacy and low adverse effects [3]. Natural products are known as one of the most important and effective drugs for human disease treatment [4–6]. In addition to their efficacy, natural products are mostly non-toxic and therefore, they can be used as safe therapeutic strategies [7–9]. A plethora of scientific evidence reported that edible and medicinal plants have significant potential to synthesize antimicrobial agents as their defense mechanisms against biotic stresses such as microorganisms [10]. It has been reported that plant-derived antimicrobial compounds can be categorized into the different groups such as phenols, flavonoids, terpenoids, lectins, polypeptides, polyacetylenes as well as alkaloids [11]. In addition, in traditional medicine, many edible and medicinal plants have been widely used for the treatment of different infectious diseases [11, 12].

The genus *Eryngium* contains more than 250 flowering species worldwide [13]. Genus *Eryngium* is the largest and most complex genus in Apiaceae family [13, 14]. Some species in the genus *Eryngium* are endangered such as *E. alpinum* L.*, E. aristulatum* Jeps.*, E. constancei* M.Y. Sheikh*, E. cuneifolium* Small*,* and *E. viviparum* J. Gay [13, 14]*.* According to the morphological studies, the genus *Eryngium* has been classified into five subgenera, including *Eryngium* subgenus *Eryngium*, *E*. subgenus *Monocotyloidea, E*. subgenus *Fruticosa*, *E.* subgenus *Semiaquatica*, and *E*. subgenus *Foetida* [15, 16]. *E.* subgenus *Eryngium* is the most common one throughout Europe, Africa and Asia, while the other subgenera are widely distributed in Australia [15, 16]. However, infrageneric analysis through sequence data of chloroplast DNA trnQ-trnK 5’-exon and nuclear ribosomal DNA ITS regions showed that there are two different subgenera including *Eryngium* and *Monocotyloidea* [17–19].

**Fig. 1:**
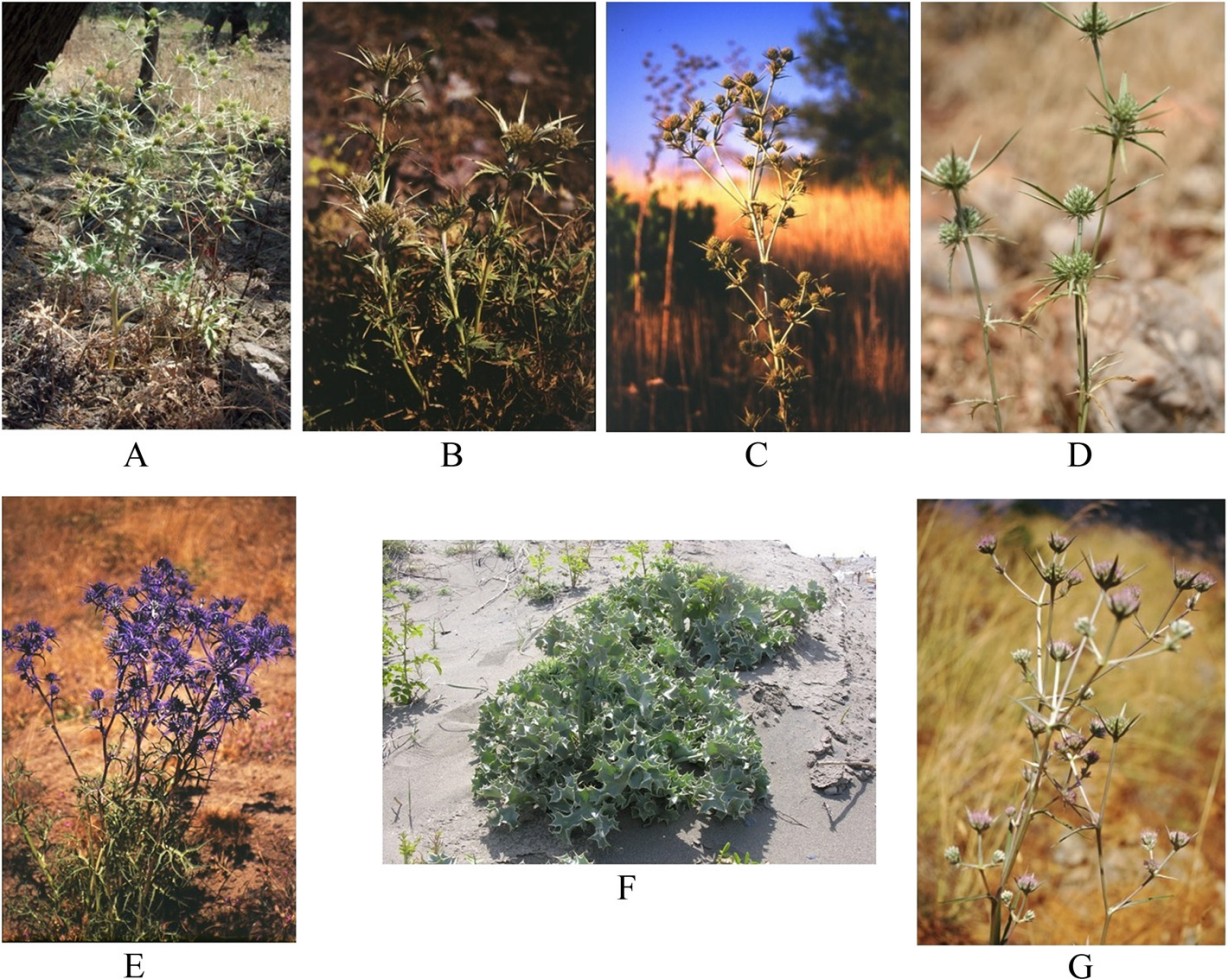
Some of species from *Eryngium* genus, *Eryngium campestre L.* (**a**), *Eryngium davisii Kit Tan & Yildiz* (**b**)*, Eryngium isauricum Contandr. & Quezel* (**c**), *Eryngium falcatum Delar.* (**d**), *Eryngium kotschyi Boiss.* (**e**), *Eryngium maritimum L.* (**f**), *Eryngium trisectum Wörz & H. Duman*. (**g**)

Like the other members of Apiaceae family, the genus *Eryngium* plants have various culinary and/or medicinal uses (Fig. 1) [20–23]. Several species have been widely used in traditional medicine such as *E. foetidum* L.*, E. caucasicum* Trautv. (syn. *Eryngium caeruleum* M. Bieb.)*, E. maritimum* L.*, E. planum* L.*, E. dichotomum* Desf*., E. campestre* L. and *E. creticum* Lam*.* [24–27]*,* whereas *E. foetidum* and *E. caucasicum* have been widely cultivated in some Asian countries such as Iran, Turkey, etc. [27, 28]. *E. caucasicum* is known as one the most common edible leafy vegetables in northern part of Iran and widely used in different foodstuff, pickles, etc. [28, 29]. Besides, the fruits of *E. foetidum* are known as common edible food components in Nigeria [30]. Actually, some *Eryngium* species are also used as ornamental plants [31]. A mountain of scientific evidence has shown that different species of the genus *Eryngium* possess antimicrobial effects under *in vitro* and *in vivo* conditions [32–36].

Therefore, the present paper aims to review the scientific literature on the phytochemical composition and antimicrobial activities of essential oils and extracts obtained from genus *Eryngium* species. In addition, we discuss about traditional uses, cultivation and phytochemistry of *Eryngium* species to provide a complete picture of this genus.

## Materials and methods

### Data sources and search strategy

Data were collected from Medline, Pubmed, Scopus, Web of Science (ISI Web of Knowledge), Science Direct, Embase, and BIOSIS Previews (from 1950 to July 20, 2015), via searching of these keywords: “*Eryngium* and phytochemistry or chemical compounds”, “*Eryngium* and antibacterial”, “*Eryngium* and antimicrobial”, and “*Eryngium* and biological effect”. We also scanned the reference list of each paper and searched Cochrane review library. Thereafter, the bibliographies of collected data were screened for further publications. Finally, collected data were analyzed and judged by second and third authors according to the scientific standard of conduct. However, some of the references may be published after the initial search date July 2015.

### Traditional uses

In traditional medicine, some of *Eryngium* species have been used for treatment of several human diseases [24–27]. It has been reported that *E. campestre* L. is widely used as antitussive, stimulant, aphrodisiac, and diuretic agent in Turkish traditional medicine [37, 38]. *E. caucasicum* is one of the most important edible plants in the northern part of Iran due to its multiple beneficial effects on human health [29, 31, 39, 40]. Moreover, *E. creticum* has been used as hypoglycemic plant in the Jordanian traditional medicine [41], while *E. elegans* Cham. & Schltdl. has been reported to be utilized as diuretic agent in the Argentinian traditional medicine [42]. Most of *Eryngium* species are also known as medicinal plants worldwide for the treatment of several human diseases such as diarrhea, gastrointestinal problems, bladder and kidney dysfunctions, and venereal diseases [24–27, 43–45]. In Chinese traditional medicine, *E. foetidum* is widely used for treatment of inflammation [24] and *E. yuccifolium* Michx. roots have been traditionally used to treat snakebites, toothache [27, 46, 47], digestive problems, diarrhea, headache, etc. [24–27, 43–45]. A large scale of evidence has pointed out to multiple pharmacological effects of the genus *Eryngium* species including antioxidant, anti-inflammatory, antihemolytic, antinociceptive effects, and protective agent against neurodegenerative deseases [25, 28, 48–50]. For instance, *Eryngium planum*, which is a rare medicinal plant, was studied to determine the effect of subchronic administration of a 70 % ethanol root extract (200 mg/kg, p.o.) on behavioral and cognitive responses in experimental animals (Wistar rats) linked with the expression levels of mRNA coding for enzymes such as acetylcholinesterase (AChE), butyrylcholinesterase (BuChE), and beta-secretase (BACE-1). At the end of the experiment, after the last dose of the *Eryngium* extract, scopolamine (SC) was administered intraperitoneally to a group of animals (treated). In the animals treated with the *Eryngium* extract, an improvement in long-term memory produced by the EP extract in both scopolamine treated and control group was registered with decreased mRNA AChE, BuChE, and BACE-1 levels, especially in the frontal cortex, suggesting the potential efficacy of this extract in this kind of pathologies [25, 28, 48–50].

This is only an example of the fact in the las decade some investigations have been carried out to demonstrate the potential pharmacological activity of *Eryngium* species extracts [51, 52].

### Cultivation

Several genus *Eryngium* species have been widely used as edible plants in many countries and, consequently, mainly cultivated as an economic crop in tropical areas of the world [24, 28, 29]. In fact, numerous reports refer to the ideal and effective conditions for cultivating, harvesting as well as post-harvesting conditions of *Eryngium* species [24, 31, 53]*.* In the northern parts of Iran, *E. caucasicum* is one of the most important garden vegetables, which is mostly used in preparation of foodstuff, pickles, etc. [28, 29]. It has been reported that some *Eryngium* species are easily cultivated in dry, sandy, well-drained soils, and full sun [24, 31, 53]. Root cuttings are a common protocol for propagation of *Eryngium* species [24, 31, 53–55], which can also be propagated using other plant cuttings [24, 31, 53–55]. In addition, there is a close correlation between *Eryngium* species growth and fertilizer levels in the soil [24, 31, 53–55]. However, *Eryngium* species are significantly endangered by plant diseases as well as insect attacks [24, 31, 53–55].

### Phytochemistry

Based on the review of the literature up to now, the aerial parts of *Eryngium* species have been reported to contain mainly saponins, flavonoids, and essential oil, while the underground parts contain triterpene saponins, monoterpene glycosides, phenolic compounds such as flavonoids and phenolic acids, coumarin derivatives, terpene aldehyde esters, acetylenes, essential oil, and oligosaccharides [56–60].

#### Saponins

**Fig. 2:**
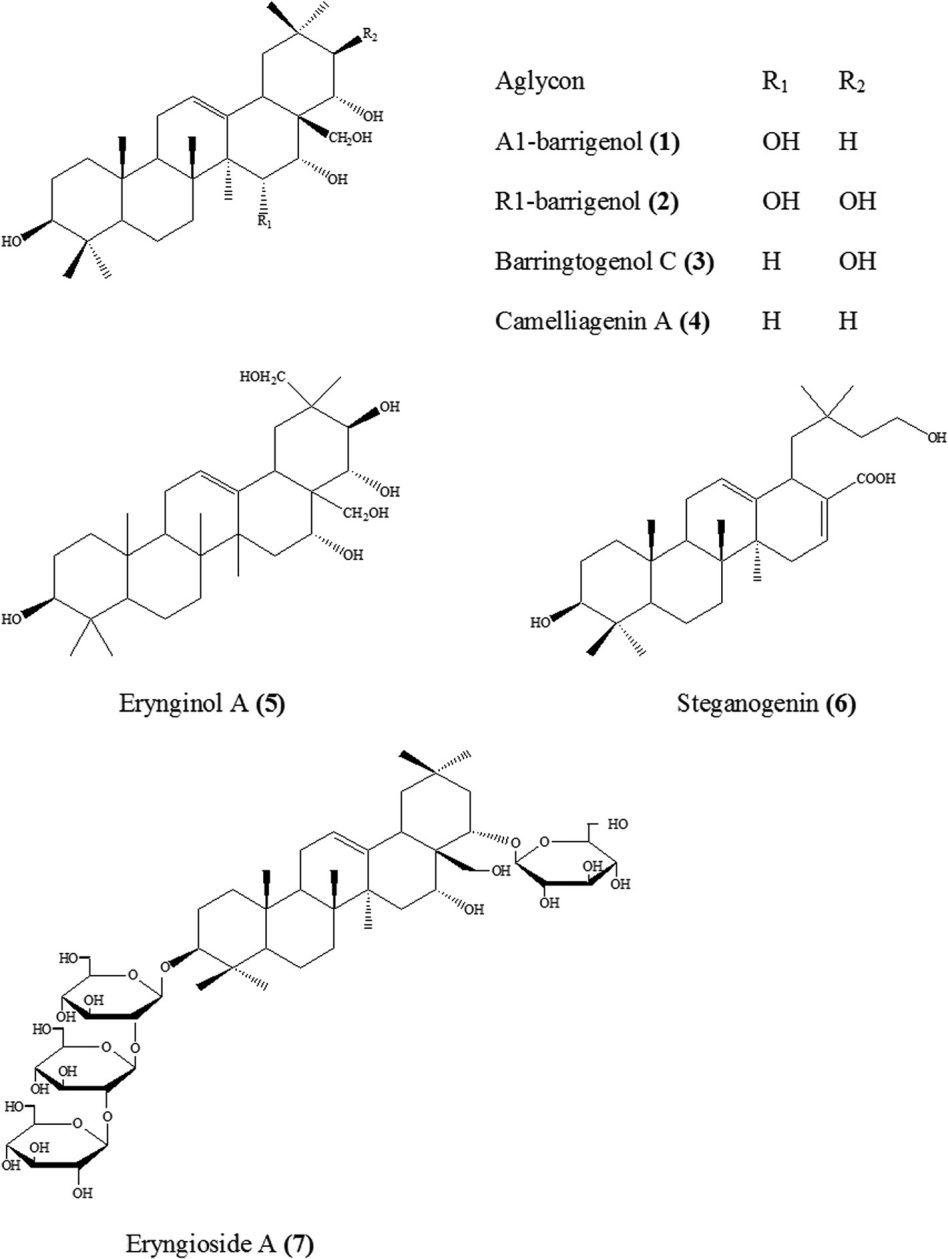
Saponin derivatives frequently found in *Eryngium* species

The *Eryngium* species are the rich sources of triterpene saponins (Tables 1 and 2). Most of the saponins isolated from *Eryngium* species possess mainly hydroxylated oleanane-type aglycons such as A1-barrigenol **(1)**, R1-barrigenol **(2)**, or barringtogenol C **(3)** (Fig. 2). In addition to these core structures, cameliagenin A **(4)**, erynginol A **(5)** and B, betulinic acid, oleanolic acid, and steganogenin **(6)** having glucose, glucuronic acid, rhamnose, xylose, galactose, and arabinose moieties have been also found in various *Eryngium* species (Fig. 2). The general saponin structures possess generally acetic, angelic, dimethylacrylic, and tiglic acid substituents, located predominantly at C21, C22 or C28 positions. On the other hand, isovaleric, *n*-butyric, and methyl butyric acids occur relatively rare substituents. Saponin glycosides found in *Eryngium* species are usually monodesmosidic saponins, where the bidesmosidic ones contain sugar groups at C3 and C28 positions [59, 61–65].

**Table 1:** Triterpene saponins from *Eryngium* species isolated between 1970–1978

Source	Sapogenol	Type of sapogenol	Plant part	Ref.
*Eryngium planum* L.	Eryngiumgenin A	A1-barrigenol	Roots	[69]
	Eryngiumgenin B	A1-barrigenol		
	Eryngiumgenin C	R1- barrigenol		
	Eryngiumgenin D	R1- barrigenol		
*Eryngium planum* L.	Erynginol A	Barringtogenol C	Aerial	[70]
*Eryngium bromeliifolium* Delar.	Oleanic acid type of sapogenols	Oleanic acid	Leaves	[66]
*Eryngium planum* L.	Eryngiumgenin F	Barringtogenol C	Roots	[61]
	Eryngiumgenin G	n/a		
	Eryngiumgenin H	n/a		
	Eryngiumgenin J	n/a		
	Eryngiumgenin K	R1- barrigenol		
	Eryngiumgenin L	n/a		
*Eryngium bromeliifolium* Delar.	Betulinic acid		Leaves	[71]
*Eryngium giganteum* L.	Giganteumgenin A	n/a	Leaves	[67]
	Giganteumgenin B	n/a		
	Giganteumgenin C	Oleanic acid		
	Giganteumgenin D	n/a		
	Giganteumgenin E	n/a		
	Giganteumgenin G	n/a		
	Giganteumgenin H	n/a		
	Giganteumgenin K	n/a		
	Giganteumgenin M	Barringtogenol C		
	Giganteumgenin N	R1- barrigenol		
*Eryngium planum* L.	Eryngiumgenin E		Roots	[72]
*Eryngium bromeliifolium* Delar.	Betulinic acid-3-*O*-*β*-glycoside		Leaves	[73]
*Eryngium maritimum* L.	Eryngiumgenin C and sapogenols with A1-barrigenol, R1-barrigenol and barringtogenol C structures		Aerial parts	[74]
*Eryngium amethystinum* L.	Main structure: Barringtogenol C			[68]
	R1-barrigenol			
	Erynginol A			
	A1-barrigenol			
*Eryngium bromeliifolium* Delar.	3-*O*-D-Glucopyranosyloleanolic acid–28-O-D-xsylopyranoside (Saponin F)		Leaves	[62]
*Eryngium planum* L.	R1-barrigenol + an acid substituent and 2 glycopyranosyl moieties		Roots	[75]^a^

^a^This study was published in 1985 and it is the only study published between 1978-2002

**Table 2:** Triterpene saponins from *Eryngium* species isolated after 2002

Source	Saponins	Plant part	Ref.
*Eryngium foetidum* (Linn)	*O*-(3)-{β-D-Glucopyranosyl-(1 → 2 rham)-β-D-fucopyranosyl-(1 → 3 rham)-α-L-rhamnopyranosyl-(1 → 4 glu)-β-D-glucopyranosyl]-olean-12-en-23,28-diol	Aerial parts	[30]
*Eryngium campestre* L.	3*-O-*β*-*D-Glucopyranosyl-(1 → 2)-[α-L-rhamnopyranosyl-(1 → 4)]-β*-*D-glucuronopyranosyl-22-*O*-Angeloyl-R1-barrigenol	Roots	[59]
	3*-O-*β-D-Glucopyranosyl -(1 → 2)-[α-L- rhamnopyranosyl -(1 → 4)]-β*-*D- glucuronopyranosyl-22-*O*-*β,β*-dimethylacryloyl-A1-barrigenol		
*Eryngium campestre* L.	3*-O-*α*-*L- Rhamnopyranosyl -(1 → 2)-β*-*D- glucuronopyranosyl -22-*O*-*β,β*- dimethylacryloyl -A1-barrigenol	Roots	[63]
	3*-O-*α*-*L- rhamnopyranosyl -(1 → 2)-β-D- glucuronopyranosyl -22-*O*- angeloyl -R1-barrigenol		
	3*-O-*α*-*L- Rhamnopyranosyl -(1 → 2)-β*-*D- glucuronopyranosyl -21-*O*-acetyl-22-*O*- angeloyl -R1-barrigenol		
	3*-O-*α*-*L- Rhamnopyranosyl -(1 → 2)-β-D- glucuronopyranosyl -21-*O*- acetyl -22-*O-β,β*- dimethylacryloyl -R1-barrigenol		
	3*-O-*α*-*L- Rhamnopyranosyl -(1 → 2)-β-D- glucuronopyranosyl -22-*O*- angeloyl -28-*O-* acetyl -R1-barrigenol		
*Eryngium yuccifolium* Michx.	3β-[β-D-Glucopyranosyl-(1 → 2)-*β*-D-glucopyranosyl-(1 → 2)]-β-D-glucopyranosyloxy-22α-*β*-D-glucopyranosyloxyolean-12-ene-16α, 28-diol (Eryngioside A)	Whole plant	[64]
	3β-[β-D-Galactopyranosyl-(1 → 2)-β-D-glucopyranosyl-(1 → 2)]-β-D-glucopyranosyloxy-22α-β-D-glucopyranosyloxyolean-12-ene-16α, 28-diol (Eryngioside B)		
	3β-[β-D-Glucopyranosyl-(1 → 2)-β-D-glucopyranosyl-(1 → 2)]-β-D-glucopyranosyloxy-22α-β-D-glucopyranosyloxyolean-12-ene-16-oxo-28-ol (Eryngioside C)		
	3β-[β-D-Glucopyranosyl-(1 → 2)-β-D-glucopyranosyl-(1 → 2)]-β-D-glucopyranosyloxy-28-β*-*D-glucopyranosyloxyolean-12-ene-16α,22α-diol (Eryngioside D)		
	22α-Angeloyloxy-3β-[β-D-glucopyranosyl-(1 → 2)]-[β-D-xylopyranosyl-(1 → 3)]-β-D- glucuronopyranosyloxyolean-12-ene-15α, 16α, 21β,28-tetrol (Eryngioside E)		
	22α-Angeloyloxy-3β-[β-D-glucopyranosyl-(1 → 2)]-[β-D-xylopyranosyl-(1 → 3)]-β-D- glucuronopyranosyloxyolean-12-ene- 16α, 21β,28-triol (Eryngioside F)		
	21*β*-Angeloyloxy-3β-[β-D-glucopyranosyl-(1 → 2)]-[α-L-arabinopyranosyl-(1 → 3)]-β-D- glucuronopyranosyloxyolean-12-ene-15α, 16α, 22*α*,28-tetrol (Eryngioside G)		
	22α-Angeloyloxy-3β-[β-D-glucopyranosyl-(1 → 2)]-[β-D-xylopyranosyl-(1 → 3)]-β-D- glucuronopyranosyloxyolean-12-ene- 15α, 16α,28-triol (Eryngioside H)		
	22α-Angeloyloxy-3β-[β-D-glucopyranosyl-(1 → 2)]-[α-L-arabinopyranosyl-(1 → 3)]-β-D- glucuronopyranosyloxyolean-12-ene- 15α, 16α,28-triol (Eryngioside I)		
	21β-Angeloyloxy-22α-acetyloxy-3β-[β-D-glucopyranosyl-(1 → 2)]-[β-D-xylopyranosyl-(1 → 3)]-β-D-glucuronopyranosyloxyolean-12-ene-15α, 16α,28-triol (Eryngioside J)		
	21β-Angeloyloxy-22α-acetyloxy-3β-[β-D-glucopyranosyl-(1 → 2)]-[β-D-xylopyranosyl-(1 → 3)]-β-D- glucuronopyranosyloxyolean-12-ene-16α,28-diol (Eryngioside K)		
	21β-Angeloyloxy-22α-acetyloxy-3β-[β-D-glucopyranosyl-(1 → 2)]-[α-L-arabinopyranosyl-(1 → 3)]-*β*-D- glucuronopyranosyloxyolean-12-ene-16α,28-diol (Eryngioside L)		
	Saniculasaponin III		
*Eryngium yuccifolium* Michx.	Eryngiosides A-L	Roots	[47]
	21 β-Acetyloxy-22 α -angeloyloxy-3 β-[β-D-glucopyranosyl-(1 → 2)]-[β-D-xylopyranosyl-(1 → 3)]-β-D-glucuronopyranosyloxyolean-12-ene-15α,16α,28-triol (Eryngioside M)		
	22α-Angeloyloxy-3β-[β-D-glucopyranosyl-(1 → 2)]-[α-L-arabinopyranosyl-(1 → 3)]-β-D- glucuronopyranosyloxyolean-12-ene-15α, 16α, 21β,28-tetrol (Eryngioside N)		
	Saniculasaponin II and III		
*Eryngium planum* L.	3-*O*-β-D-Glucopyranosyl-(1 → 2)-β-D-glucuronopyranosyl-21-*O*-acetyl-22-*O*-angeloyl-R1-barrigenol	Roots	[76]
	3-*O*-β-D-Glucopyranosyl-(1 → 2)-β-D-glucuronopyranosyl-22-*O*-angeloyl-A1-barrigenol		
	3-*O*-β-D-glucopyranosyl-(1 → 2)-β-D-glucuronopyranosyl-22-*O*-angeloyl-R1-barrigenol		
*Eryngium kotschyi* Boiss.	3-*O*-α-L-Rhamnopyranosyl-(1 → 4)-β-D-glucuronopyranosyl-22-*O*-β,β-dimethylacryloylA1-barrigenol	Roots	[65]
	3-*O*-α-L-Rhamnopyranosyl-(1 → 4)-β-D-glucuronopyranosyl-22-*O*-angeloylA1-barrigenol		
	3-*O*-β-D-glucopyranosyl-(1 → 2)-[β-D-glucopyranosyl-(1 → 6)]-β-D-Glucopyranosyl-21,22,28-*O*-triacetyl-(3β,21β,22α)-olean-12-en-16-one		
	3-*O*-β-D-Glucopyranosyl-(1 → 2)- glucopyranosyl-22-*O*-β-D- glucopyranosylsteganogenin		
	3-*O*-β-D-Galactopyranosyl-(1 → 2)-[α-L-arabinopyranosyl-(1 → 3)]-β-D-glucuronopyranosyl-22-*O-*angeloyl-A1-barrigenol		
	3-*O*-α-L-Rhamnopyranosyl-(1 → 4)-β-D-glucuronopyranosyloleanolic acid

**Fig. 3:**
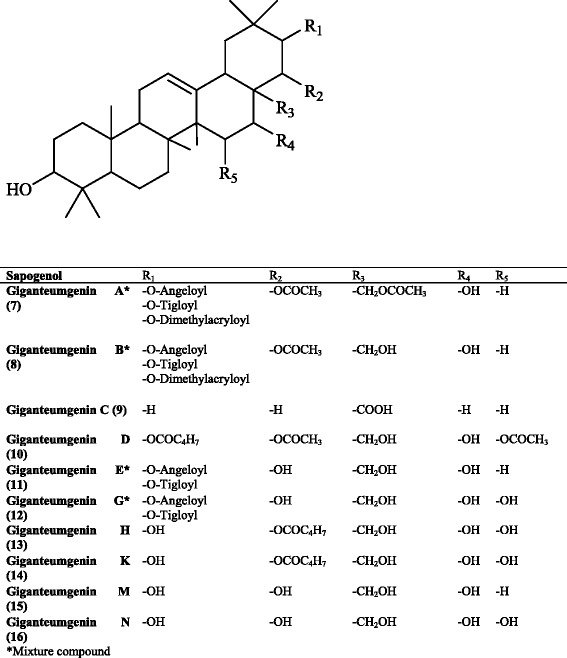
Structures of giganteumgenins A-N

The first phytochemical studies performed on the genus *Eryngium* starting from early 1970s were focused on their saponin content. According to the survey of the literature data published between 1970–1978 and in 1985, the identification of the isolated compounds was limited to the absolute definition of the aglycons with possible positions of sugars and acyl substituents because of inadequate chemical facilities in structure elucidation techniques [61, 66–68]. In this period, *E. planum, E. amethystinum* L., *E. giganteum* M. Bieb., and *E. bromeliifolium* F. Delaroche were studied in more detail. As summarized in Table 1, A1-barrigenol and R1-barrigenol-type of aglycons containing dimethylacrylic, angelic, and/or tyglic acids as the acid moieties were isolated from the roots of the *E. planum*, named as eryngiumgenine A-D [69] of which only aglycon types were identified and classified according to the R_f_ values. Later, compound **(5)** was isolated from the aerial parts of this species [70]. Further phytochemical studies on the same plant were continued on the leaves and roots. These studies led to the isolation of a number of aglycons with **(4)**, **(3)**, **(2)** types possessing acetic, tyglic, butyric, and isovalerianic acids located at C16, C21, C22 or C28 positions [71, 72]. Isolation of saponin aglycones such as **(2)** and **(3)**, and saponins bearing **(2)** and **(3)** skeletons, named as giganteumgenin A **(7)**, B **(8)**, C **(9)**, D **(10)**, E **(11)**, G **(12)**, H **(13)**, K **(14)**, M **(15)**, and N **(16)** (Fig. 3), was achieved from the methanol extract of the leaves of *E. giganteum* [67]. Another phytochemical investigation on *E. amethystinum* led to isolation of the saponins with main structures in **(1)**, **(2)**, **(3)**, and **(5)** [68]. Other sapogenols isolated from various *Eryngium* species until 1978 are listed in Table 1 [58, 62, 72–74]. Many years later, another R1-barringtogenol derivative with an acid moiety and two glycopyranosyl moieties was reported from *E. planum* roots by Voigt et al. in 1985 [75].

**Fig. 4:**
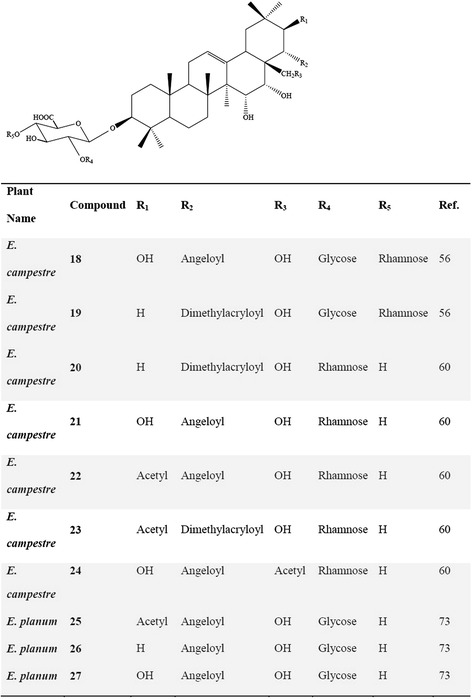
Structure of the (17) isolated from *E. foetidum*

**Fig. 5:**
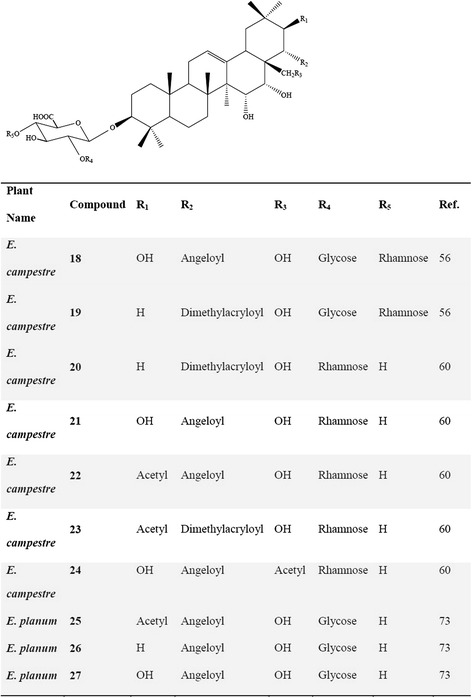
Saponins isolated from *E. campestre* and *E. planum*

**Fig. 6:**
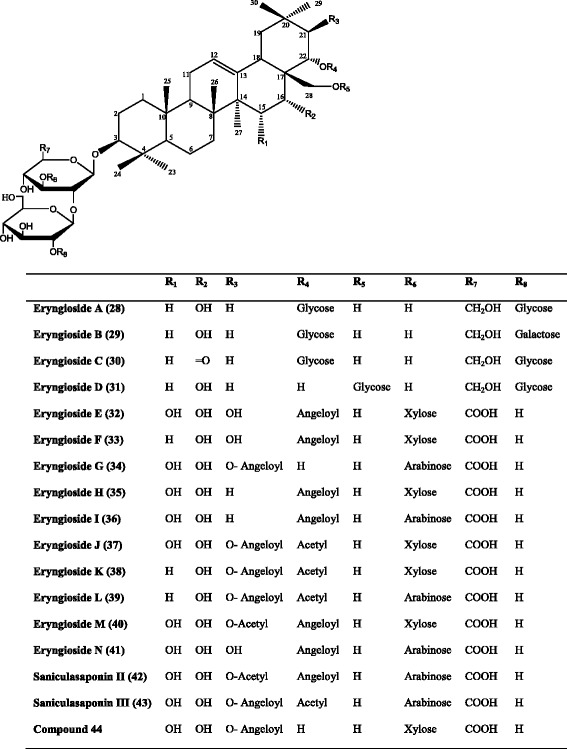
Saponins isolated from *E. yuccifolium*

Most of the scientific articles concerning saponins of the genus *Eryngium* remained limited to the identification of the sapogenol type and determination of the acid or sugar substituents. After the late 70’s, publications concerning the saponins from the genus *Eryngium* have become much more detailed as giving the whole structure elucidations as a result of the developments of the techniques used for structure elucidation. For instance, Anam [30] reported the full configuration of a new oleanane-type triterpene saponin structure **(17)** from the aerial parts of *E. foetidum* (Fig. 4). Kartal et al. [59] characterized two new triterpene saponins **(18, 19)** from the roots of *E. campestre* bearing **A(1)** and **(2)** types, and isolated five new triterpene saponins **(20–24)**, one of which was **(1)** and the others with **(2)** main skeleton (Fig. 5) [63]. Another phytochemical study performed on the whole parts of *E. yuccifolium* was reported by Zhang et al. [64], who described the isolation and identification of 12 new polyhydroxylated triterpenoid saponins named as eryngiosides A-L **(28–39)** and two known triterpenoid saponins {21β-angeloyloxy-3β-[β-D-glucopyranosyl-(1 → 2)]-[β-D-xylopyranosyl-(1 → 3)]-β-D-glucuronopyranosyloxyolean-12-ene-15α, 16α, 22α, 28-tetrol **(44)** and saniculasaponin III **(43)**} from the same species (Fig. 6). Furthermore, the root of *E. yuccifolium* afforded two new polyhydroxyoleanene saponins [eryngioside M **(40)** and N **(41)**], together with 15 known triterpenoid saponins **(28–39; 42–44)** elucidated [47].

**Fig. 7:**
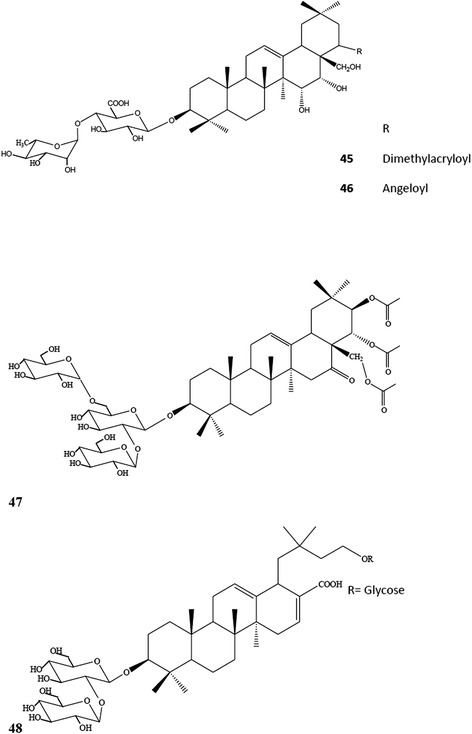
Chemical structure of triterpene saponins

Further studies on the roots of *E. planum* led to the isolation of two R1- and one A1-barrigenol type of triterpene saponins [76], where 3-O-β-D- glucopyranosyl-(1 → 2)-β-D-glucuronopyranosyl-21-O-acetyl-22-O-angeloyl-R1-barrigenol **(25)**, 3-O-β-D-glucopyranosyl-(1 → 2)-β-d-glucuronopyranosyl-22-O-angeloyl-A1-barrigenol **(26)** and 3-O-β-D-glucopyranosyl-(1 → 2)-β-D-glucuronopyranosyl-22-O-angeloyl-R1-barrigenol **(27)** (Fig. 5) were also found to be present in the roots of *E. planum* [76]. Detailed phytochemical investigation on the roots of *E. kotschyi* Boiss., which is an endemic plant to Turkey, led to isolation of two known and four new triterpene saponins **(45–48)** (Fig. 7) [77].

#### Flavonoids

**Fig. 8:**
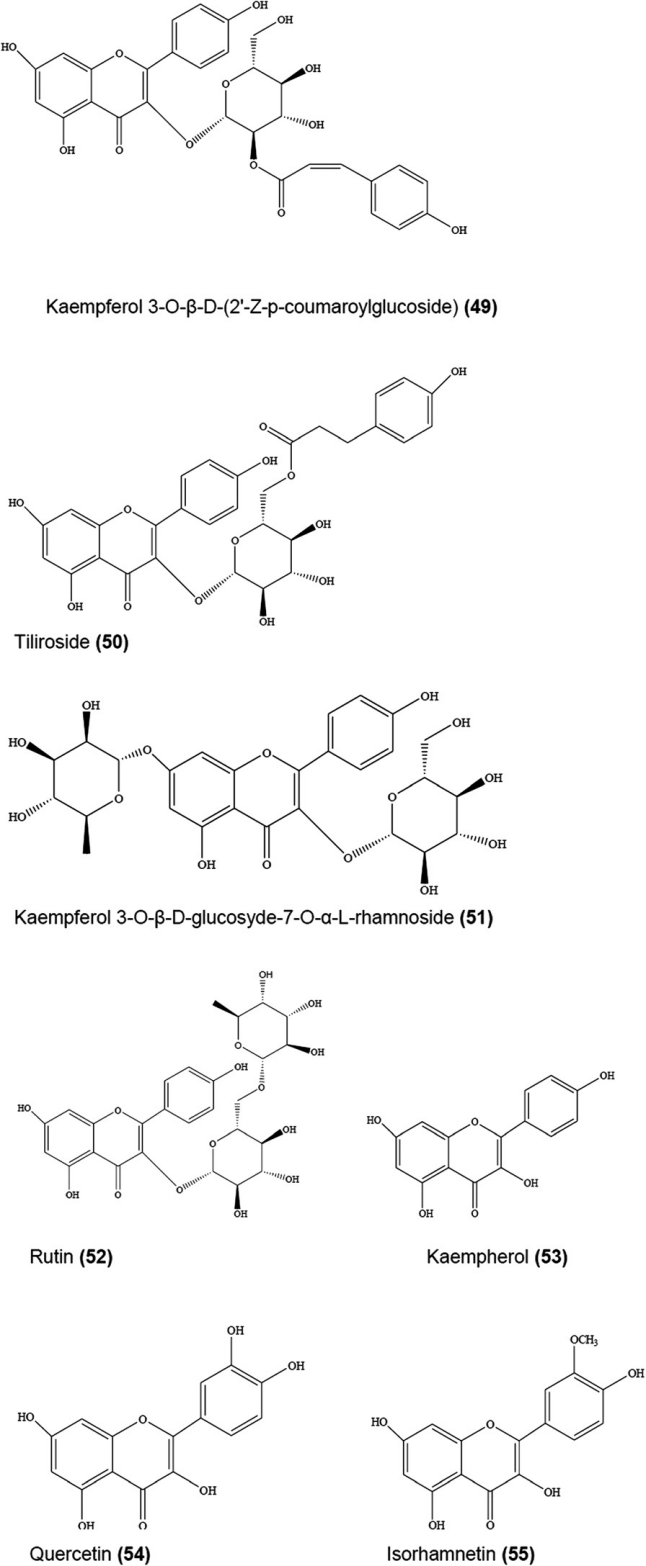
Selected flavonoids (49–55) isolated from *E. campestre*

In an earlier study [78], isolation from *E. planum* of a new flavonol glycoside (identified as kaempferol-3-O-(6-O-β-D-glucopyranosyl)-β-D-galactopyranoside) was reported. A phytochemical study on the aerial parts of *E. campestre* led to the characterization of a new acylated flavonol named as kaempferol 3-*O*-β-D-(2’-*Z*-*p*-coumaroylglucoside) **(49)** along with 10 known compounds including tiliroside **(50)**, kaempferol 3-O-β-D-glucosyde-7-O-α-L-rhamnoside **(51)**, rutin **(52)**, kaempferol **(53)**, quercetin **(54)**, isorhamnetin **(55)**, caffeic acid, chlorogenic acid, and mannitol [58] as well as luteolin 7-glucoside [79] of which some selected structures are given in Fig. 8. Using UHPLC-ESI-Q-TOF-MS technique, quantification of a number of flavonols (quercetin, kaempferol, isorhamnetin, and their derivatives) and naringenin rhamnoglucoside (a flavanone derivative) was performed in *E. bourgatii* Gouan by Cádiz-Gurrea et al. [48]. Hawas et al. [80] isolated 11 flavonoid glycosides (including isorhamnetin 3-O-α-rhamnoside, isorhamnetin 3-O-β-galactoside, isorhamnetin 3-O-β-glucoside, isorhamnetin 3-O-β-rutinoside, myricetin 3-O-β-galactoside 4’-methylether, myricetin 3-O-β-glucoside 3’-methylether, myricetin 3-O-β-glucoside 4’-methylether, quercetin 3-O-β-glucoside, quercetin 3-O-β-glucuronide 4’-methylether, and rutoside) from the aerial parts of *E. campestre*. Then, most recently, Khalfallah et al. [36] characterized five flavonoid derivatives from the aerial parts of *E. triquetrum* Vahl. described as kaempferol 3-*O*-β-D-glucoside, kaempferol 3-*O*-[6´´-*O*-*E*-*p*-coumaroyl]-β-D-glucopyranoside, kaempferol 3-*O*-[2´´,6´´-di--*O*-*E*-*p*-coumaroyl]-β-D-glucoside, kaempferol 3-*O*-[α-L-rhamnosyl-(6 → 1)-*O*-β-D-glucoside, and quercetin 3-*O*-[α-L-rhamnosyl-(6 → 1)-*O*-β-D-glucoside].

#### Coumarin derivatives

**Fig. 9:**
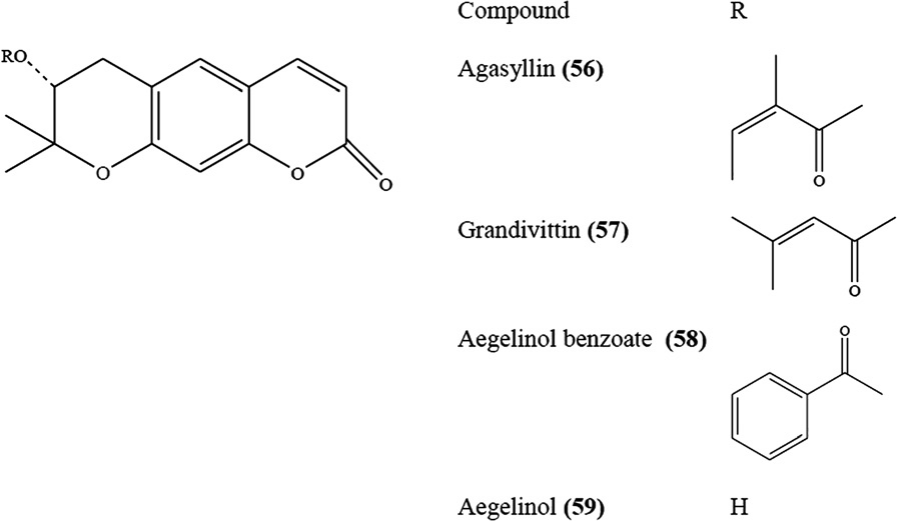
Some coumarin derivatives isolated from *E. campestre*

**Fig. 10:**
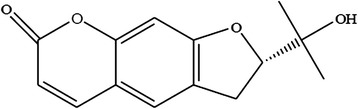
Nodakenetin (60) isolated from *E. ilicifolium*

The first coumarin derivatives isolated from *E. campestre* by Sticher & Erdelmeier [56] were agasyllin **(56)**, grandivittin **(57)**, aegelinol benzoate **(58)**, and aegelinol **(59)** which structures are given in Fig. 9 [56]. The isolation of marmesin tiglate [also known as nodakenetin **(60),** Fig. 10] together with metetoin, a nitrogenous compound, was achieved from *E. ilicifolium* Lam. [81].

#### Phenolic acid derivatives

**Fig. 11:**
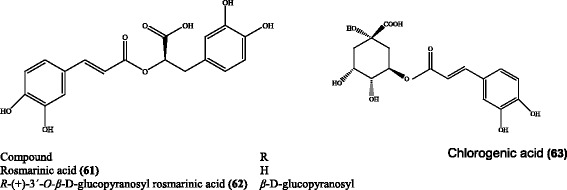
Some phenolic acid derivatives found in *E. alpinum*

Many *Eryngium* species have been reported to contain phenolic substances. Le Claire et al. [82] characterized chlorogenic **(63)**, R-(+)-rosmarinic **(61)**, and *R*-(+)-3’-*O-β*-D-glucopyranosyl rosmarinic acids **(62)** in the roots of *E. alpinum* through medium pressure liquid chromatography (MPLC) and preparative high pressure liquid chromatography (HPLC) (Fig. 11). Besides, the presence of R-(+)-rosmarinic acid and *R*-(+)-3’-*O-β*-D-glucopyranosyl rosmarinic acid was also shown in several *Eryngium* species which could be used as chemotaxanomic markers specific to this genus. Two new caffeic acid derivatives, i.e. 3,4-dihydroxyphenyl caffeate and (4-β-D-glucopyranosyloxy)-3-hydroxyphenyl caffeate, along with a new flavonoid (kaempferol-3-O-(2-O-*trans*-*p*-methoxycoumaroyl-6-O-*trans*-*p*-coumaroyl)-β-D-glucopyranoside) were isolated from the whole parts of *E. yuccifolium* in addition to following known compounds, i.e. caffeic acid and kaempferol-3-O-(2,6-di-O-*trans*-*p*-coumaroyl)-β-D-glucopyranoside [64]. Cádiz-Gurrea et al. [48] also revealed presence of cinnamic acid derivatives (chlorogenic, rosmarinic, ferulic, caffeic acids, and their derivatives), benzoic acid derivatives (*p*-hydroxybenzoic acid glucoside, arbutin, syringic acid, glucogallin, gentisic acid, and their derivatives) and various organic acids (gluconic, citric, quinic acids, and their derivatives) in *E. bourgatii* using hyphenated advance liquid chromatographic separation techniques. On the other hand, catechin, epicatechin, chlorogenic, gallic, and rosmarinic acids were determined quantitatively in the roots of *E. palmatum* Pančić & Vis. [83]. In a similar study [60], chlorogenic, hydroxybenzoic, and caftaric acids were detected in *E. bornmuelleri* Nábělek along with trace amounts of caffeic, ferulic, and rosmarinic acids.

#### Other types of compounds

Drake and Lam [84] reported the isolation of falcarinone, a widespread acetylenic compound occurring in Apiaceae. 6-Pentyl-2-[2-oxo-butin(3)-yliden]-tetrahydropyrane from the aerial parts and roots of *E. bourgatti* and the acetylenic compounds identified as *Z* and *E* isomers of the same compound were also obtained from this species in addition to falcarinone, falcarinolone, falcarinol, and scopoletin [85]. Further phytochemical studies ensued in the characterization of two new monoterpene glycosides of the cyclohexenone type, elucidated as 3-(β-D-glucopyranosyloxymethyl)-2,4,4-trimethyl-2,5-cyclohexadien-1-one and 3-(β-D-glucopyranosyloxymethyl)-2,4,4-trimethyl-2-cyclohexen-1-one [57]. An unusual sesquiterpene whose structure was established as 1-*n*-propyl-perhydronaphthaline 1,2,4a,5,6,7,8,8a-octahydro-4-methyl-1-propyl-naphthalene-7-carbaldehyde was reported from the aerial parts of *E. creticum* [86], while new ester derivatives, characterized as *cis*-chrysanthenyl hexanoate and *cis*-chrysanthenyl octanoate, were isolated from *E. planum* [87].

In order to find out the compounds possibly responsible for the anti-inflammatory activity of *E. foetidum*, composition of the hexane extract from the leaves of this species was subjected to GC and GC-MS analyses [88]. Hereby, α-cholesterol, brassicasterol, campesterol, stigmasterol (as the main component, 95 %), clerosterol, β-sitosterol, ∆_5_-avenasterol, ∆_5_24-stigmastadienol, and ∆_7_-avenasterol were detected in the extract. Muckensturm et al. [89] reported a phytochemical study performed on the diethyl ether extracts from *E. giganteum*, *E. variifolium* Coss.*, E. planum*, and *E. maritimum.* Chromatographic separation techniques applied to the seed extract of *E. giganteum* led to the elucidation of a new *nor*-sesquiterpene hydrocarbon, i.e. 15-*nor*-α-muurolene (gigantene), and germacrene-D, *trans*-β-farnesene, 15-oxy-α-muurolene, 15-hydroxy-α-muurolene, ledol, and spatulenol. Similar studies on the seeds of the *E. planum* gave way to isolation of *cis*-chrysanthenyl acetate, while isoferulyl senecioate and 2,3,4-trimethylbenzaldehyde were obtained from the leaves of *E. variifolium*. Nevertheless, it should be noted that the latter compound was considered as artifact by these authors. In another study [90], *E. foetidum* was subjected to isolation procedures which finally afforded *trans*-2-dodecanal (eryngial) in pure form.

#### Essential oil

Essential oil compositions of numerous *Eryngium* species have been investigated by many researchers. As illustrated with their corresponding references in Table 3, a great interspecies variation could be easily observed. However, the most common monoterpenes analyzed in different plant parts of *Eryngium* species have been as follows; germacrene D, α-pinene, caryophyllene, muurolene, α- and β-selinene, limonene, α- and β-bisabolol, etc., whereas many hydrocarbons and some aromatics such as trimethylbenzaldehyde and dodecenal have been detected.

**Table 3:** The major components in the essential oils of various *Eryngium* species

Plant Name	Plant part	Major components	Ref.
*Eryngium alpinum* L.	Aerial parts	Caryophyllene oxide (21.6 %)	[98]
		Bicyclogermacrene (11.8 %)	
		Germacrene D (10.3 %)	
*Eryngium amethystinum* L.	Aerial parts	*β*-Caryophyllene (19.7 %)	[98]
		*α*-Bisabolol (7.9 %)	
		2,3,6-Trimethylbenzaldehyde (7.9 %)	
	Leafy parts of the shoots	*α*-Pinene (11.8 %)	[99]
		2,3,6-Trimethylbenzaldehyde (24.7 %)	
		Germacrene D (31.3 %)	
	Inflorescence	*α*-Pinene (25.6 %)	
		2,3,6- Trimethylbenzaldehyde (22.0 %)	
		Germacrene D (14.5 %)	
	Fruit	*α*-Pinene (17.0 %)	
		2,3,6-Trimethylbenzaldehyde (16.9 %)	
		Germacrene D (7.6 %)	
*Eryngium billardieri* F. Delaroche	Aerial parts	*α*-Muurolene (42.0 %)	[39]
		*β*-Gurjunene (17.0 %)	
		*δ*-Cadinene (6.2 %)	
		Valencene (5.7 %)	
*Eryngium bourgatii* Gouan	Inflorescence	Phyllocladene (37.6 %)	[13]
		Bicyclogermacrene (15.1 %)	
	Stems & leaves	Phyllocladene (20.4 %)	
		*γ*-Muurolene (11.8 %)	
		(*E*)-Caryophyllene (10.1 %)	
	Roots	*γ*-Muurolene (15.4 %)	
		Phyllocladene (15.0 %)	
*Eryngium bungei* Boiss.	Aerial parts	Cumin alcohol (55.3 %)	[100]
		Terpinolene (14.6 %)	
		Carvacrol (8.9 %)	
		Limonene (7.5 %)	
	Aerial parts	Borneol (44.4 %)	[101]
		Isobornyl formate (14.7 %)	
		Isoborneol (9.2 %)	
		1,8-Cineol (9.1 %)	
		Camphor (7.9 %)	
*Eryngium caeruleum* M.B.	Aerial parts	Limonene (60.5 %)	[102]
		δ-3-Carene (13.0 %)	
	Aerial parts	Cyclobuta[1–4]dicycloocten Hexadecahydro (47.03 %)	[35]
		*n*-Hexadecanoic acid (11.16 %)	
		Limonene (4.23 %)	
		*Cis*-α-bisabolene (2.14 %)	
*Eryngium campestre* L.	Inflorescence	Germacrene D (30.3–40.3 %)	[103]
		β-Curcumene (0.7–22.2 %)	
		Myrcene (3.0–21.7 %)	
		(*E*)-β-Farnesene (0.1–19.0 %)	
	Stems & leaves	Germacrene D (31.1–42.4 %)	
		Myrcene (0.5–23.15 %)	
*Eryngium caucasicum* Trautv.	Leaves (coastal samples)	4(5)-Acetyl-1H-imidazole (63.6 %)	[104]
		Thymol (13.9 %)	
		β-Sesquiphellandrene (10.0 %)	
	Leaves (hill slope samples)	β-Sesquiphellandrene (44.3 %)	
		Limonene (20.1 %)	
		*Trans*-β-Farnesene (14.1 %)	
*Eryngium corniculatum* Lam.	Inflorescence	2,4,6-Trimethylbenzaldehyde (50.8 %)	[105]
		*α*-Pinene (4.0 %)	
		Crystanethylacetate (4.0 %)	
		2,4,5-Trimethylbenzaldehyde (%3.3)	
	Stems & leaves	2,4,6-Trimethylbenzaldehyde (50.0 %)	
		2,4,5-Trimethylbenzaldehyde (3.8 %)	
	Roots	2,4,6-Trimethylbenzaldehyde (29.8 %)	
		Phyllocladene isomer (13.0 %)	
		(*E*)-Nerolidol (9.4 %)	
*Eryngium creticum* Lam.	Flowering aerial parts	Hexanal (52.9 %)	[37]
		Heptanal (13.9 %)	
		Octane (8.95 %)	
*Eryngium duriaei* subsp. *juresianum* (M. Laínz) M. Laínz	Aerial parts	α-Neocallitropsene (26.0 %)	[106]
		Isocaryophyllen-14-al (16.2 %)	
		14-Hidroxy-β-caryophyllene (13.4 %)	
		Caryophyllene oxide (7.6 %)	
		*E*-β-Caryophyllene (6.3 %)	
*Eryngium expansum* F. Muell.	Aerial parts	7-*Epi*-Selinene (38.3 %)	[107]
		*Cis*-β-Guaiene (10.8 %)	
		2,3,6-Trimethylbenzaldehyde (8.0 %)	
*Eryngium foetidum* L.	Aerial parts	2,3,6-Trimethylbenzaldehyde (5.5–23.7 %)	[108]
		(E)-2-Dodecenal (15.9–37.5 %)	
		(E)-2-Tetradecenal (18.7–25.3 %)	
	Aerial parts	2,4,5-Trimethylbenzaldehyde (27.7 %)	[109]
		*(E)*-2-Dodecenal (27.5 %)	
		Carotol (8.8 %)	
		3-Dodecenal (5.2 %)	
	Aerial parts	(*E*)-2-Dodecenal (57.79–67.08 %)	[110]
		Lauraldehyde (7.04–11.53 %)	
		13-Tetradecenal (8.99–9.03 %)	
	Leaves	2,4,5-Trimethylbenzaldehyde (20.53 %)	[111]
		Hexadecanoic acid (12.05 %)	
		Carotol (9.94 %)	
	Roots	Duraldehyde (37.60–53.14 %)	[110]
		13-Tetradecenal (7.22–13.16 %)	
		(*E*)-2-Dodecenal (7.14–11.62 %)	
		Falcarinol (3.44–8.06 %)	
*Eryngium glaciale* Boiss.	Inflorescence	Phyllocladene isomer (43.5 %)	[112]
		(*E*)-Caryophyllene (15.2 %)	
		Valencene (11.5 %)	
	Stems & leaves	Phyllocladene isomer (41.3 %)	
	Roots	Phyllocladene isomer (49.4 %)	
		Linalool (19.1 %)	
*Eryngium maritimum* L.	Aerial parts	Spathulenol (18.99 %)	[77]
		Caryophyllene oxide (8.18 %)	
	Aerial parts	Germacrene D (10.4 %)	[113]
		2,4,5-Trimethylbenzaldehyde (8.3 %)	
	Roots	Germacrene D (15.9 %)	
		2,4,5-Trimethylbenzaldehyde (6.7 %)	
	Aerial parts	Germacrene D (13.7–45.9 %)	[114]
		4βH-Cadin-9-en-15-al (18.4–27.6 %)	
		4βH-Cadin-9-en-15-ol (2.2–14.3 %)	
		4βH-Muurol-9-en-15-al (4.3–9.3 %)	
*Eryngium pandanifolium* Cham. et Schlecht	Leaves	Bornyl acetate (20.8 %)	[107]
		β-Selinene (13.8 %)	
		α-Selinene (11.3 %)	
	Fruit	Octanal (11.5 %)	
		β-Selinene (9.2 %)	
*Eryngium palmatum*	Roots	Octanal (31.7 %)	[115]
		Curcumene (5.9 %)	
		2,3,6-Trimethylbenzaldehyde (5.4 %)	
*Eryngium paniculatum* Cav.	Inflorescence	(*E*)-Anethole (52.6 %)	[116]
		α-Pinene (19.1 %)	
*Eryngium planum* L.	Inflorescence	*Cis*-Chrysanthenyl acetate (43.2 %)	[117]
	Stalk leaves	Limonene (14.7 %)	
		β-Pinene (9.8 %)	
	Rosette leaves	Bornyl acetate (18.1 %)	
		Limonene (11.3 %)	
		Terpinen-4-ol (10.9 %)	
	Roots	Falcarinol (64.4 %)	
*Eryngium rostratum* Cav.	Stem	Spathulenol (20.0 %)	[107]
		β-Bisabolol (8.6 %)	
		Caryophyllene oxide (8.0 %)	
	Fruit	γ-Terpinene (4.5 %)	
		α-Muurolene (3.9 %)	
*Eryngium rosulatum* P. W. Michael ined.	Aerial parts	β-Elemene (16.0 %)	[118]
		Bicyclogermacrene (12.5 %)	
		δ-Elemene (7.0 %)	
*Eryngium thorifolium* Boiss.	Flowering aerial parts	(1R)-α-Pinene (58.6 %)	[37]
		Limonene (3.14 %)	
*Eryngium tricuspidatum* L.	Aerial parts	α-Bisabolol (32.6 %)	[34]
		α-Curcumene (6.5 %)	
*Eryngium vesiculaosum* Labill.	Winter leaves	β-Caryophyllene (20.3 %)	[119]
		Germacrene D (19.2 %)	
		α-Humulene (8.8 %)	
	Summer leaves	Bicyclogermacrene (22.2 %)	
		β-Caryophyllene (15.6 %)	
		Germacrene D (15.8 %)	
		α-Humulene (8.1 %)	
*Eryngium yuccifolium* Michaux.	Leaves	Germacrene D (18.3 %)	[120]
		Terpinolene (17.8 %)	
		Bicyclogermacrene (8.8 %)	
		α-Pinene (7.6 %)	
		β-Caryophyllene (6.2 %)	
		Falcarinol (9.6 %)	
	Stalks	Germacrene D (38.4 %)	
		γ-Amorphene (12.2 %)	
		Bicyclogermacrene (10.1 %)	
		Bicyclosesquiphellandrene (3.4 %)	
		Falcarinol (3.2 %)	
	Roots	Terpinolene (25.8 %)	
		*Trans*-β-Bergamotene (18.6 %)	
		Benzaldehyde 2,3,6-trimethylbenzaldehyde (13.9 %)	

### Antimicrobial effects of the genus *Eryngium* plants

Plants are known to produce antimicrobial substances [10], which act as plant defense mechanisms and protect them against abiotic and biotic stresses. These antimicrobial agents, which are often characterized by low adverse effects and wide spectrum activities, belong to many chemical classes such as phenolics and polyphenolics, terpenoids, alkaloids, lectins, polypeptides, and polyacetylenes [11].

Among the plants belonging to the genus *Eryngium*, some species exhibit considerable antimicrobial activity against gram-positive and gram-negative bacteria, some species of fungi and yeasts and viruses. Within this genus, the most studied species has been *E. foetidum*, which is cultivated across South Asia and Europe, Tropical Africa, and Pacific islands. As regards the antibacterial activity of *E. foetidum* extracts, the literature data are conflicting. In fact, in 2003, Alzoreky and Nakahara reported that the acetone and buffered methanol extracts obtained from *E. foetidum* leaves did not show any antibacterial activity against *Escherichia coli*, *Salmonella infantis*, *Listeria monocytogenes* Tottori, *Staphylococcus aureus* and *Bacillus cereus* [91].

More recently, Ndip et al. showed that the methanolic extract form *E. foetidum* leaves showed moderate antibacterial activity against 6 clinical strains of *Helicobacter pylori* out of 15 tested strains, using the disk diffusion technique as antibacterial susceptibility test [92]. Besides antibacterial activity, *E. foetidum* was tested for its antiplasmodial activity, using chloroquine as positive control to evaluate the sensitivity of susceptible *Plasmodium falciparum* strains. The leaf extracts were prepared using hexane, dichloromethane, and methanol to obtain three extracts. The dried extracts were then dissolved in DMSO to give a stock solution at 10 mg/mL, used for the biological tests. The results indicated that *E. foetidum* showed low in vitro antiplasmodial activity against *P. falciparum,* with an IC50 value of 25 *μ*g/mL [93].

Another species belonging to genus *Eryngium,* is *E. maritimum*, which is a wild perennial species growing on sand beaches in West Europe, the Mediterranean basin, and the Black Sea and has been used for its diuretic, stimulant, cystotonic, stone inhibitor, aphrodisiac, expectorant, and anthelmintic properties (http://www.botanicals.com, http://www.crescentbloom.com). The essential oil, obtained from hydrodistillation of the aerial parts, was found to contain a known sesquiterpene (muurol-9-en-15-al) and three new oxygenated sesquiterpenes with a muurolane or cadinane skeleton (4βH-cadin-9-en-15-al, 4βHmuurol-9-en-15-ol, and 4βH-cadin-9-en-15-ol), The sesquiterpenoid-rich fraction was tested in vitro using the agar diffusion method and the minimum inhibitory concentration (MIC) in the liquid phase against *L. monocytogenes* and *E.coli*. The finding revealed that the antibacterial activity of the new oxygenated sesquiterpenes against the tested bacteria, with an inhibition diameter higher than 15 mm and a MIC value lower than 90 μg/mL [94].

Another investigation on *E. maritimun* showed that the leaf hydromethanolic extract fractionated into a polar (aqueous) and apolar (chloroformic) fraction and tested using the microdilution method against food-borne pathogens and clinical isolates, exhibited antimicrobial activity. The tested Gram-positive bacteria were *S. aureus* subsp. *aureus, Micrococcus luteus, L. monocytogenes* and *B. cereus.* The Gram-negative bacteria were two strains of *Salmonella* (*S. enterica* subsp. *arizonae* and *S. enterica* subsp. *montevideo*)*,* three strains of *Pseudomonas* (*P. aeruginosa, P. fluorescens,* and *P. marginalis*)*, E. coli,* and *Erwinia carotovora* subsp. *carotovora,* and a yeast (*Candida albicans*). According to the data obtained, the fractions resulted to be active against all bacteria with the exception of *L. monocytogenes*, while the most sensitive bacteria were *P. aeruginosa* and *P. fluorescens*, with MIC values of 1 and 2 μg/mL for the polar and apolar fractions, respectively [95].

The essential-oil composition and antimicrobial activity of three other species belonging to genus *Eryngium* (*E. creticum, E. campestre*, and *E. thorifolium*), whose infusions obtained from the aerial and root parts are commonly used in Turkish folk medicine as antitussive, diuretic, stimulant, and aphrodisiac, were studied [96]. The composition of *E. thorifolium* was found to be rich in α-pinene, a known antibacterial terpenic compound, which was present in fewer amounts in the other two species. Differently, *E. creticum* was found to be rich in hexanal, which was present in less amount in *E. thorifolium* and was not detected in *E. campestre.* Antibacterial activity of the essential oils was tested with the disc diffusion method against nine clinical strains of methicillin-resistant *S. aureus* (MRSA). The essential oil obtained from *E. thorifolium*, which caused an inhibition zone ranging from 13 to 19 mm (similar to that exhibited by vancomycin and oregano essential oil tested at 10 μL/disc and 5 μL/disc, respectively), was demonstrated to be the most active species [37]. As aforementioned, many *Eryngium* species, e.g. *E. maritimum* exerted antifungal activity against *C. albicans* and other strains. Especially, the essential oil obtained by water distillation of the aerial parts of *E. duriaei* Gay ex Boiss subsp. *juresianum* (M.Laínz) M. Laínz was tested for its antifungal activity against 13 fungi, among which there were seven dermatophyte species (*Microsporum canis* FF1, *Trichophyton mentagrophytes* FF7, *Epidermophyton floccosum* FF9, *M. gypseum* CECT 2905*, T. rubrum* CECT 2794, *T. mentagrophytes* var interdigitale CECT 2958 and *T. verrucosum* CECT 2992), five *Candida* species (two clinical isolates from recurrent cases of vulvovaginal or oral candidosis, *C. krusei* (H9) and *C. guillermondii* MAT23, along with three reference species of *C. albicans* ATCC 10231, *C. tropicalis* ATCC 13803, and *C. parapsilosis* ATCC 90018) and a strain of *Cryptococcus neoformans* CECT 1078. Using the macrodilution broth method the MIC and Minimal Lethal Concentrations (MLC) were determined. The results disclosed that the essential oil did not possess antifungal activity against *Candida* strains, with the exception of *C. guillermondii*, which resulted to be sensitive to the antifungal activity of the oil with a MIC and MLC of 2.5 μL/mL. On the contrary, the essential oil had MIC values of 0.16–0.32 μL/mL against all the tested dermatophyte species. The essential oil was further studied to determine its chemical composition by GC-MS and the authors suggested that caryophyllene derived compounds [isocaryophyllen-14-al (16.2 %), 14-hidroxy-β-caryophyllene (13.4 %), caryophyllene oxide (7.6 %) and *E*-β-caryophyllene (6.3 %)] are probably the responsible for the antifungal activity [97].

Finally, in 2013, the antiphytoviral activity of the essential oils obtained by water distillation of the aerial parts of *E. alpinum* and *E. amethystinum* cultivated in Croatia was described. The antiviral activity was shown in *Chenopodium quinoa* treated with the essential oils (250 ppm) prior to the inoculation of cucumber mosaic virus associated with a satellite RNA. The number of leaf local lesions, registered in the presence of the essential oils, was strongly reduced from an average value of 14.9 ± 0.8 to 3.3 ± 0.2 and 2.9 ± 0.2, for *E. alpinum* and *E. amethystinum* essential oils*,* respectively. The authors ascribed the antiviral activity to the occurrence of caryophyllene oxide and β-caryophyllene, which were identified as the major components of *E. alpinum and E. amethystinum*, respectively. Moreover, other components such as germacrene D, α-bisabolol, and γ-eudesmol, which were detected as minor oil constituents, might be responsible for the inhibition of viral infection [98].

The summary of the literature data was reported in Table 4.

**Table 4:** Antimicrobial activity of *Eryngium* species extracts

*Eryngium* plant	Type of extract	Used method	Microbes	Positive control	Ref.
*E. foetidum*	Methanolic extract of leaves	disk diffusion technique	6 clinical strains of *Helicobacter pylori*	ns^a^	[92]
*E. foetidum*	hexane, dichloromethane, and methanol extracts	disk diffusion technique	*Plasmodium falciparum*	chloroquine	[93]
*E. maritimum*	hydrodistillation of the aerial parts	agar diffusion method and minimum inhibitory concentration (MIC)	*L. monocytogenes E. coli*.	ns^a^	[94]
*E. maritimum*	hydromethanolic extract of leaves	microdilution method	*S. aureus* subsp. *aureus, Micrococcus luteus, B. cereus. S. enterica* subsp. *arizonae S. enterica* subsp. *Montevideo P. aeruginosa, P. fluorescens, P. marginalis E. coli Erwinia carotovora* subsp. *carotovora, Candida albicans*	ns^a^	[95]
*E. creticum, E. campestre*, *E. thorifolium*	essential oils from aerial and root parts	disk diffusion method	9 clinical strains of methicillin-resistant *S. aureus* (MRSA)	ns^a^	[37]
*E. duriaei*	essential oil obtained by water distillation of the aerial parts	macrodilution broth method	*Microsporum canis* FF1, *Trichophyton mentagrophytes* FF7, *Epidermophyton floccosum* FF9, *M. gypseum* CECT 2905*, T. rubrum* CECT 2794, *T. mentagrophytes* var interdigitale CECT 2958 *T. verrucosum* CECT 2992 *Candida. guillermondii* MAT23, *Cryptococcus neoformans* CECT 1078.	ns^a^	[97]
*E. alpinum*, *E. amethystinum*	essential oils obtained by water distillation of the aerial parts	count of leaf local lesions in *Chenopodium quinoa* treated with the essential oils prior to the inoculation of virus	cucumber mosaic virus associated with a satellite RNA	ns^a^	[98]

^a^not specified

## Conclusion

The present paper shows that essential oils and extracts obtained from various *Eryngium* species have broad range antimicrobial activity against several strains of gram-positive and gram-negative bacteria, some species of fungi and yeasts, and viruses. The findings point out to the fact that these activities can be ascribed to the presence of different phytochemicals, especially apolar compounds. However, our search at https://clinicaltrials.gov/ with keyword “*Eryngium*” accessed on February 9, 2015 showed no clinical trial regarding the beneficial effects of the genus *Eryngium* plants against different types of infection available up to date. In addition, there are only a few papers on the toxicity of the active constituents belonging to the genus *Eryngium* plants, which resulted to be cytotoxic especially at high doses. It can be suggested that more toxicity studies should be carried out prior to the clinical trials.

According to this study, we conclude that the essential oils and extracts of those *Eryngium* species that have been submitted to in vitro investigation (Table 4) should be proceeded to toxicological studies and *in vivo* experiments as multi-target antimicrobial agents for the treatment of human infectious diseases, especially antibiotic-resistant bacterial infections. Thus, it is not quite possible to make a clear statement or comment about their clinical uses. Hence, we recommend that future studies should be performed on:

- toxicity of the *Eryngium* plant extracts and essential oil resulted to be active in in vitro experiments.
- clinical studies of the safe extracts of *Eryngium* species with in vitro activity,
- finding the exact mechanism underlying the antibacterial effects of the essential oils and extracts of members of the genus *Eryngium* and their antibacterial constituents,
- separation, isolation, and structure identification of the most antibacterial constituents of the essential oils and extracts of different members of the genus *Eryngium* and their interactions with foods as well as common synthetic antibacterial compounds,
- ascertaining the most effective and safe doses for clinical studies regarding the antibacterial effects of the essential oils and extracts of different genus *Eryngium* species against different infectious diseases.
